# Biomagnetic of Apatite-Coated Cobalt Ferrite: A Core–Shell Particle for Protein Adsorption and pH-Controlled Release

**DOI:** 10.1007/s11671-010-9761-4

**Published:** 2010-08-31

**Authors:** I-Ming Tang, Nateetip Krishnamra, Narattaphol Charoenphandhu, Rassmidara Hoonsawat, Weeraphat Pon-On

**Affiliations:** 1 ThEP Center, Commission of Higher Education, 328 Si Ayuthaya Rd., Bangkok 10400, Thailand; 2 Consortium for Calcium and Bone Research, Faculty of Science, Mahidol University, Bangkok 10400, Thailand; 3 Department of Physics, Faculty of Science, Mahidol University, Bangkok 10400, Thailand; 4 Department of Physiology, Faculty of Science, Mahidol University, Bangkok 10400, Thailand; 5 Mahidol University International College, Salaya Campus, Mahidol University, Salaya 73720, Thailand

**Keywords:** Protein release, Cobalt ferrite, Apatite, Bovine serum albumin (BSA)

## Abstract

Magnetic nanoparticle composite with a cobalt ferrite (CoFe_2_O_4_, (CF)) core and an apatite (Ap) coating was synthesized using a biomineralization process in which a modified simulated body fluid (1.5SBF) solution is the source of the calcium phosphate for the apatite formation. The core–shell structure formed after the citric acid–stabilized cobalt ferrite (CFCA) particles were incubated in the 1.5 SBF solution for 1 week. The mean particle size of CFCA-Ap is about 750 nm. A saturation magnetization of 15.56 emug^-1^ and a coercivity of 1808.5 Oe were observed for the CFCA-Ap obtained. Bovine serum albumin (BSA) was used as the model protein to study the adsorption and release of the proteins by the CFCA-Ap particles. The protein adsorption by the CFCA-Ap particles followed a more typical Freundlich than Langmuir adsorption isotherm. The BSA release as a function of time became less rapid as the CFCA-Ap particles were immersed in higher pH solution, thus indicating that the BSA release is dependent on the local pH.

## Introduction

The targeting of biomolecules (e.g., drug, DNA, or protein) using delivery vehicles that are biocompatible with the human body is an area of great interest. To combine into one vehicle, two types of materials, a magnetic material that has the magnetic property needed for the targeting and a biopolymer that possess a biocompatibility with the human body, would be of a great advantage to the treatment of human illnesses [1-6]. Magnetic composites can also be used in magnetic bioseparation [7-12], drug delivery [13-16], and hyperthermia [17-22] applications. The particles in all of these applications should have a core/shell structure, with the core being the magnetic material. The core must be pre-coated with a shell to insure their stability, biodegradability, and non-toxicity in the physiological medium [23-27]. These shells are often made with biopolymers. The shell can also be made with inert materials such as calcium phosphate of apatite. Hydroxyapatite has an excellent biocompatibility with the human body and degrades slowly in physiological medium (pH ~ 7.4) [28-31]. Apatite is also an ideal material for the surface coating of the core since its surface is porous that allows for the adsorption of biological molecules. This is very important since the purification and separation of protein and enzymes are achieved by attaching (functionalizing) chemical radicals that have an affinity for these biomolecules for the nanoparticles.

In this study, we are interested in vehicles (magnetic nanocomposites) that can be guided to their targets by the action of a magnetic field. The targeting of the biomolecules would lessen the possible toxic effects the biomolecules may have on the human body when too much of the biomolecules are introduced into the human body. The toxic effects would also be lessened by the coating. The coating may however lead to changes in the composition, size, morphology, and surface chemistry of the magnetic particles. These changes in turn may lead to changes in the magnetic behavior of core material in vivo. The response of the magnetic core to an external field should be the same after the coating.

Fe_3_O_4_ (magnetite) was the magnetic material used [1-6,15-20] in the initial applications of magnetic nanoparticles. Another magnetic material which also has been used is CoFe_2_O_4_ (cobalt ferrite) [32-34]. Cobalt ferrite has a higher magneto-crystalline anisotropy that results in it having a higher coercivity field, *H*_c_ (the field at which the magnetization is zero). Importantly, it possesses good chemical stability. This is needed since the functionalization of magnetic nanoparticle by the carboxylic groups is performed in a mild acidic solution. In this study, cobalt ferrite was therefore chosen to be the core material.

Composites of magnetic particles and calcium phosphate have been studied already. Wu et al. [35] showed that the calcium phosphate of hydroxyapatite-based magnetic nanoparticles has good biocompatibility with the human body and that it does not elicit any cytotoxicity. Pareta et al. [36] showed that in the presence of bovine serum albumin (BSA) protein, the osteoblast density of magnetic particles coated with calcium phosphate increased significantly after 1 day of immersion. The BSA is one of the most abundant proteins found in blood plasma. Its high content of carboxyl (–COO^-^) and amino (–NH_3_^+^) groups makes it a useful tool in the study of the adsorption of proteins.

We report here a method for synthesizing apatite-coated citric acid–stabilized cobalt ferrite particles (CFCA-Ap) that have a core–shell structure. Its structure, morphology, chemical stability, and magnetic properties of products have been investigated. BSA was used as a model protein to study the adsorption and sustained release of proteins from the CFCA-Ap particles in simulated protein delivery experiments.

## Experimental

Apatite (Ap)-coated citric acid (CA) stabilizes cobalt ferrite (CoFe_2_O_4_) (CFCA-Ap) particles that were fabricated in a two-step process. (1) Carboxylic groups from citric acid (COO^-^) were used to stabilize the surface of the magnetic cobalt ferrite (CF). This was done by immersing the CF in 0.001 M citric acid. At this point, citric acid–stabilized cobalt ferrite (CFCA) particles were formed (CF suspension in aqueous solution). (2) For the biomimetic process, the CFCA particles were then immersed in a simulated body fluid (SBF) solution. The anionic surface of CFCA particles accelerated the chelating of the calcium ions present in the SBF solution onto the surface of the CFCA. These then react with the phosphate ions (also in the SBF) to form the apatite layer on the cobalt ferrite surface. These became the apatite-coated critic acid–stabilized cobalt ferrite (CFCA-Ap) core–shell particles. The individual steps in the fabrication process are as follows:

### Preparation of the Cobalt Ferrite (CoFe_2_O_4_) (CF) Nanoparticles

The cobalt ferrite nanoparticles were prepared using NaOH, Fe(NO_3_)_3_ · 9H_2_O and Co(NO_3_)_2_ · 6H_2_O obtained from UNIVAR (Australia). The sodium dodecyl sulfate (SDS), Na_2_SO_4_, and ethylene glycol (EG) were obtained from Fluka (Switzerland). The ratio of Fe to Co was kept at 2:1 mol%. Each chemical constituent was dissolved in 25 ml de-ionized water. These solutions were then mixed together. This was then added to a solution of 3 M ethylene glycol (5 ml) and 0.001 M Na_2_SO_4_ (3 ml). The pH of the resulting CoFe_2_O_4_ precipitate was adjusted by adding NaOH under constant stirring conditions. The temperature at which the reaction occurred was kept at 60°C throughout. After adding the NaOH to the solution, the formation of black particles of cobalt ferrite could be seen. After the completion of the reaction, the solution was continuously stirred for another 30 min. The cobalt ferrite powder was obtained by centrifuge and freeze drying.

### Formation Core–Shell Structure of Apatite-Coated Cobalt Ferrite

**Figure 1:**
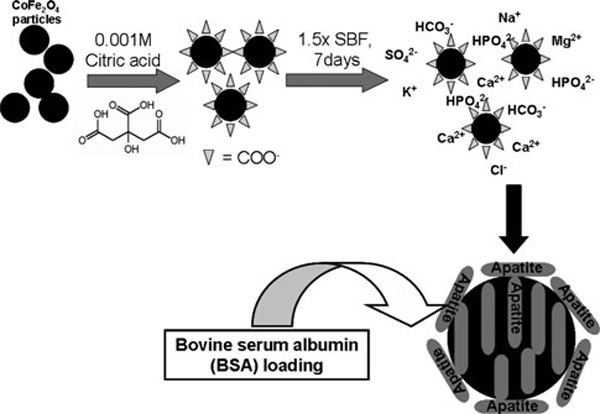
**Flowchart for the apatite (Ap)-coated citric acid–stabilized cobalt ferrite (CFCA-Ap) synthesis via biomineralization from 1.5SBF solution method and BSA protein-loaded CFCA-Ap particles**.

The procedure for the synthesis of citric acid-functionalized CoFe_2_O_4_ particles (CFCA) is shown in Figure 1. First, the CoFe_2_O_4_ particles are synthesized as outlined earlier. These CoFe_2_O_4_ particles are then dispersed in 0.001 M of citric acid (concentration 50 mg/ml) (UNiVAR, Australia) and stirred overnight. At the end of this stage, the samples consist of cobalt ferrite stabilized with carboxylic group (–COO^-^) and are labeled as CFCA. The suspension is separated by a permanent magnet and the resulting composite powder was freeze-dried. The citric acid–stabilized cobalt ferrite particles are coated by apatite following their immersion in the modified simulated body fluid (1.5SBF). The incubation was achieved by immersing the CFCA powders in 50 ml of 1.5SBF for 1 week at 37°C under static conditions. At this stage, the apatite layer was gradually formed on the CFCA surface. These particles are referred to as CFCA-Ap. After completing the immersion in the 1.5SBF, the particles were washed with de-ionized water and freeze-dried before any further analysis.

The SBF is a fluid having an ionic composition very similar to that of human plasma [37,38]. The SBF solution we used is one of the more extensively used solution. It consists of the following chemicals: NaCl (136.8 mM), NaHCO_3_ (4.2 mM), KCl (3.0 mM), K_2_HPO_4_ (1.0 mM), MgCl_2_ · 6H_2_O (1.5 mM), CaCl_2_ (2.5 mM) and Na_2_SO_4_ (0.5 mM). These chemicals are mixed together with the pH adjusted to 7.4. The SBF is replaced every 3 days to avoid any changes in the cationic concentration that may occur due to the degradation of the sample.

### Materials Characterization

The crystal structures of the powders were determined by powder X-ray diffraction (XRD) (Bruker diffractometer, Model D8 Advance) using the Cu K_α_ radiation and operating at 40 kV with 40 mA current. The XRD patterns were scanned from 2*θ* = 20°–60° at a scanning speed of 1 s per step with an increment of 0.037° per step. For the FT-IR absorption measurements, the powders were mixed with KBr and pressed into pellets using a pressure of 10 tons for 1 min. The pellets were analyzed using a FT-IR spectrophotometer (Spectrum GX, Perkin Elmer) that performed 16 scans over the range 370–4,000 cm^-1^. The magnetic properties of CFCA-Ap particles were measured using a room temperature VSM (vibrating sample magnetometer (Lakeshore, Model 4500)). The coercivities (*H*_c_), the remnant magnetizations (*M*_r_), and the saturation magnetizations (*M*_s_) of the samples were measured with the VSM. The room temperature magnetic parameters (*H*_c_, *M*_r_, and *M*_s_) of each sample were determined from the hysteresis loops produced by the VSM. A scanning electron microscope (SEM) (JEOL model JSM-6301F) was used to observe the size and morphology of the samples. An accelerating voltage of 15 kV was used to obtain the SEM images. The formation of apatite crystals on the cobalt ferrite surface was analyzed with an energy dispersive spectroscopy (EDS) (ISIS 300 (Oxford Instruments)). The core–shell particles were also examined by a transmission electron microscope (TEM) (JEOL model JEM-2010). The electron diffraction attachment to the TEM was used to obtain electron diffraction patterns of CFCA-Ap particles. Particle size distribution and zeta-potential measurement of CFCA-Ap were measured with a Zetasizer Nano ZS (Malvern, UK).

## Results and Discussion

### Results of Characterization Studies

**Figure 2:**
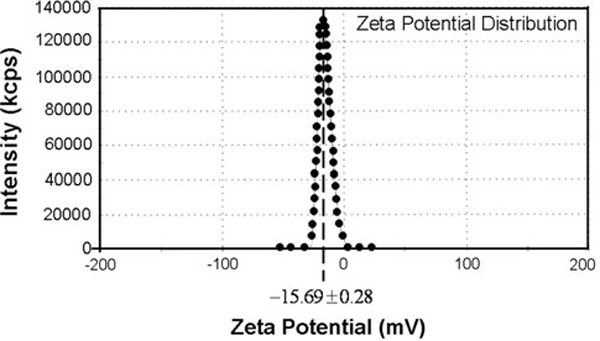
**Zeta-potential of citric stabilize cobalt ferrite (CFCA) particles**.

**Figure 3:**
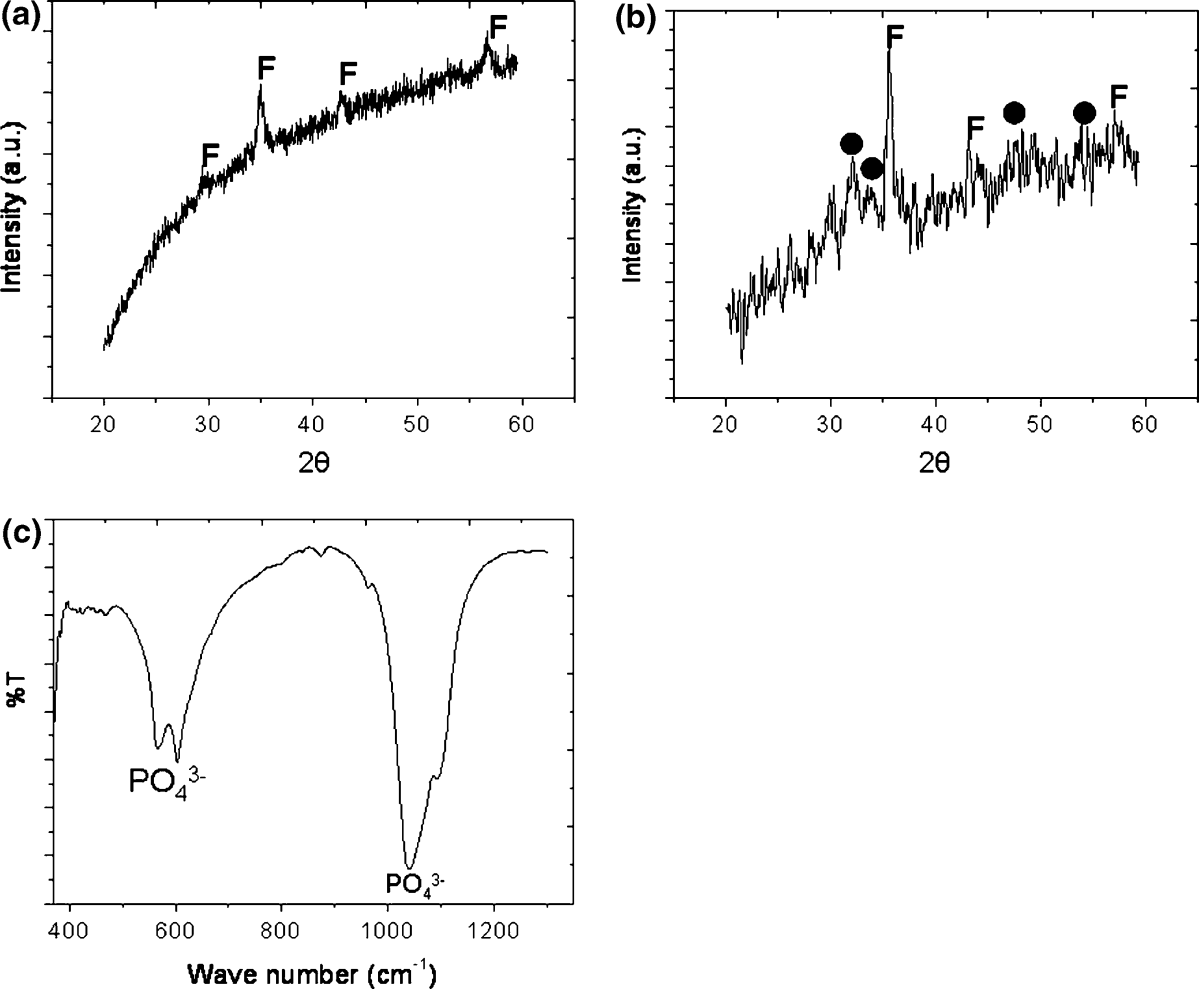
**The XRD pattern of CoFe_2_O_4_ (CF) (a), apatite (Ap)-coated CFCA particles (CFCA-Ap) (b), and FTIR spectra of CFCA-Ap particles (c)**. *Filled circle*, hydroxyapatite phase and ***F***, ferrite phase.

The formation of the calcium phosphate layer on the CFCA surface during the immersion of the CFCA particles in the SBF solution involves both the nucleation and growth of inorganic apatite mineral. The negative charge on the CFCA particles causes the calcium and phosphate ions present in the 1.5SBF solution to accumulate on the surfaces of the CFCA particles. On Figure 2, we have plotted the zeta-potential (ζ) of CFCA particle measured with the Zetasizer Nano ZS. It gives a negatively charged surface value of -15.69. The XRD patterns and FT-IR spectrums of the particles are shown in Figure 3. Figure 3a shows the diffraction pattern of the CF particle. The diffraction peaks at the Bragg angles at 30.2°, 35.7°, 43.6°, 53.6°, and 57.3° (represented by **F** letter) are the reflection peaks from the (220), (311), (400), (511), and (440) lattice planes, which are the peaks of cobalt ferrite [22,23,33,34]. The XRD pattern of CFCA after being incubated in SBF is shown in Figure 3b. The patterns of cobalt ferrite and the peak pattern of calcium phosphate of hydroxyapatite (HAp) [28,29] at 2*θ* ~ 26°, 28°, and 30–32° are seen. Evidence of hydroxyapatite formed on the CFCA particles can be seen in the FT-IR reflection spectra. The FT-IR spectrums of CFCA-CaP are shown in Figure 3c. The characteristic bands of PO_4_^3-^ groups in the apatite can be seen in the FT-IR spectrum of the CFCA-Ap particles. The intense bands at 1,088, 1,035, and 961 cm^-1^ are due to the PO_4_^3-^ stretching modes, while the doublet at 602 and 562 cm^-1^ are due to the PO_4_^3-^ bending mode [29]. The shoulder signals around 1,450 and 1,240 cm^-1^ and the doublet peaks around 873 cm^-1^ are characteristics of carbonated ions substituted into the phosphate site in an apatite structure, the so-called B-type apatite [39].

**Figure 4:**
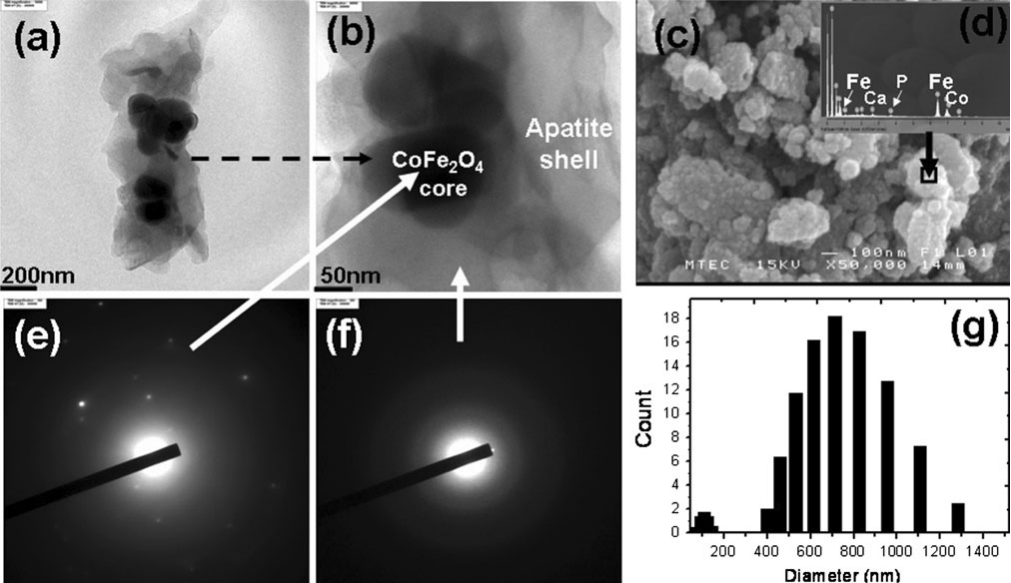
**TEM image of core–shell structure of apatite (Ap)-coated citric acid–stabilized cobalt ferrite (CFCA-Ap) in low (a) and high-resolution field (b), and SEM image of CFCA-Ap particles (c)**. EDS analysis of the samples at the CFCA-Ap surface: Ca/P = 1.47 (**d**). Core–shell structure of CFCA-Ap particles corresponding electron diffraction patterns of core structure (**e**) and shell structure (**f**). **g** is size distribution of core–shell structure of CFCA-Ap.

The TEM image of CFCA-Ap shows the core–shell structure (Figure 4a, b). The calcium phosphate of hydroxyapatite coating on the CFCA surface is seen to be inhomogeneous. Plates like structure are distributed around the spherical CF particle. The composite particles formed have an average core of 100–200 nm and the shell thickness is about 50–100 nm. An analysis of the SEM images of the CFCA-Ap particles (Figure 4c) provides some insight into the mechanism of nucleation. They show the growth of apatite mineral to be the aggregation of particles to form globular spheres less than 1 μm in size. Large peaks that appear in the EDS patterns from the surface of the CFCA-Ap (seen in the insert (Figure 4d)) are due to Fe and Co to Ca and P. These peaks indicate that the average atomic Ca/P ratio is 1.47, which is within the range seen in biological apatite [28,29]. The ED patterns of CFCA-Ap particle at the core (Figure 4e) are indicative of the polycrystalline nature of the cobalt ferrite particles. They show that the particles are composed of fine randomly orientated crystal grains. The lower intensity of the diffraction peaks emanating from the embedded CoFe_2_O_4_ core is due to the decrease in the intensity of the incident electron before it reaches the core. The electron diffraction ring patterns of the apatite at the surface of cobalt ferrite particles are shown in Figure 4f. The pattern emanating from the shell exhibits poorly resolved ring spot due to the disordered nature of the materials in the shell. The distribution in the sizes of the particles is shown in Figure 4g. The diameters of the particles are in the range between 400 and 1,200 nm, with a mean particle diameter of about 750 nm. The relative high diameter implied that a high degree of agglomerations had occurred. This may have resulted from the heterogeneous surface activities of the mineralization processes.

**Figure 5:**
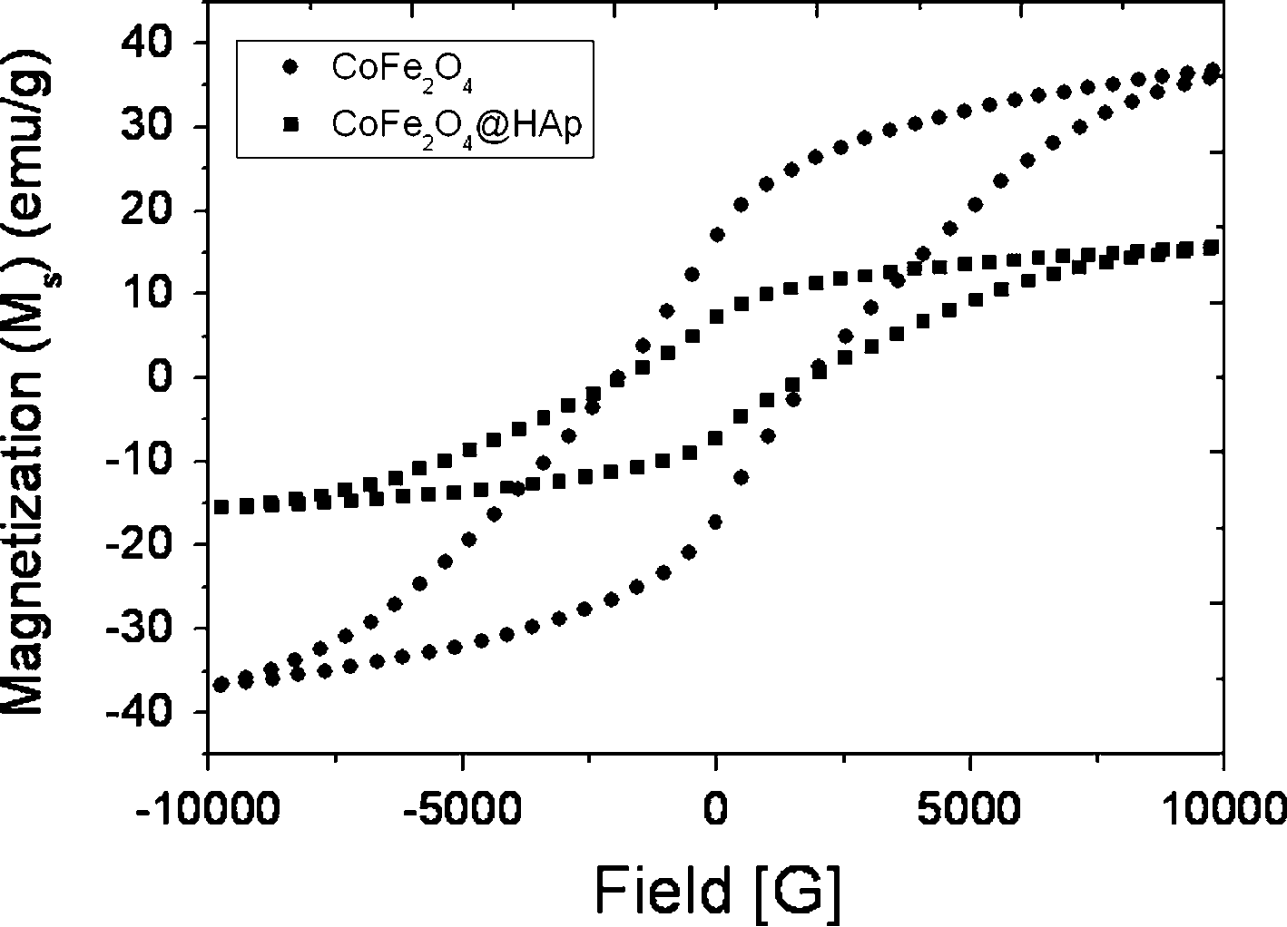
**Hysteresis loops obtained from a VSM measured operating at room temperature for CoFe_2_O_4_, and apatite (Ap)-coated citric acid–stabilized cobalt ferrite (CFCA-Ap) particles**.

The room temperature magnetic properties of CF and CFCA-Ap were recorded under an applied field of 10 kOe. The samples exhibit hysteresis loops typical of magnetic materials having an ordered magnetic structure (Figure 5). The saturation magnetizations (*M*_s_) derived from the hysteresis loops of cobalt ferrite particles are higher than those of apatite-coated cobalt ferrite particles. The coercivity (*H*_c_) of the CoFe_2_O_4_ is slightly lower after coated by apatite. The values of the *H*_c_ are 1896.9 and 1808.5 Oe for the CF and CFCA-Ap particles, respectively. The lower value of magnetization for CFCA-Ap compared with that CF is due the lower amount of the magnetic particles in the coated composites. Another interpretation is that the non-magnetic coating layer (apatite) forms a magnetic dead layer on the surface. This will affect the magnitude of the magnetization by quenching the surface moment that contributes to surface anisotropy.

### Adsorption and Release of BSA from CFCA-Ap Particles

Various amounts of BSA (Fluka, Switzerland) were dissolved in phosphate buffer saline (PBS) with a pH of 7.4 to obtain final concentrations of 0.2, 0.4, 0.6, 0.8, 1.0, and 1.2 mg/ml. Twenty-five milligrams of the CFCA-Ap particles was mixed for 4 h with 5 ml of each BSA concentration using a mildly magnetic stirrer. The temperature was kept at 37°C throughout the experiment. At the conclusion of this mixing, the protein-loaded CFCA-Ap powders were removed from the liquid by a permanent magnet. The supernatants were kept for later use.

**Figure 6:**
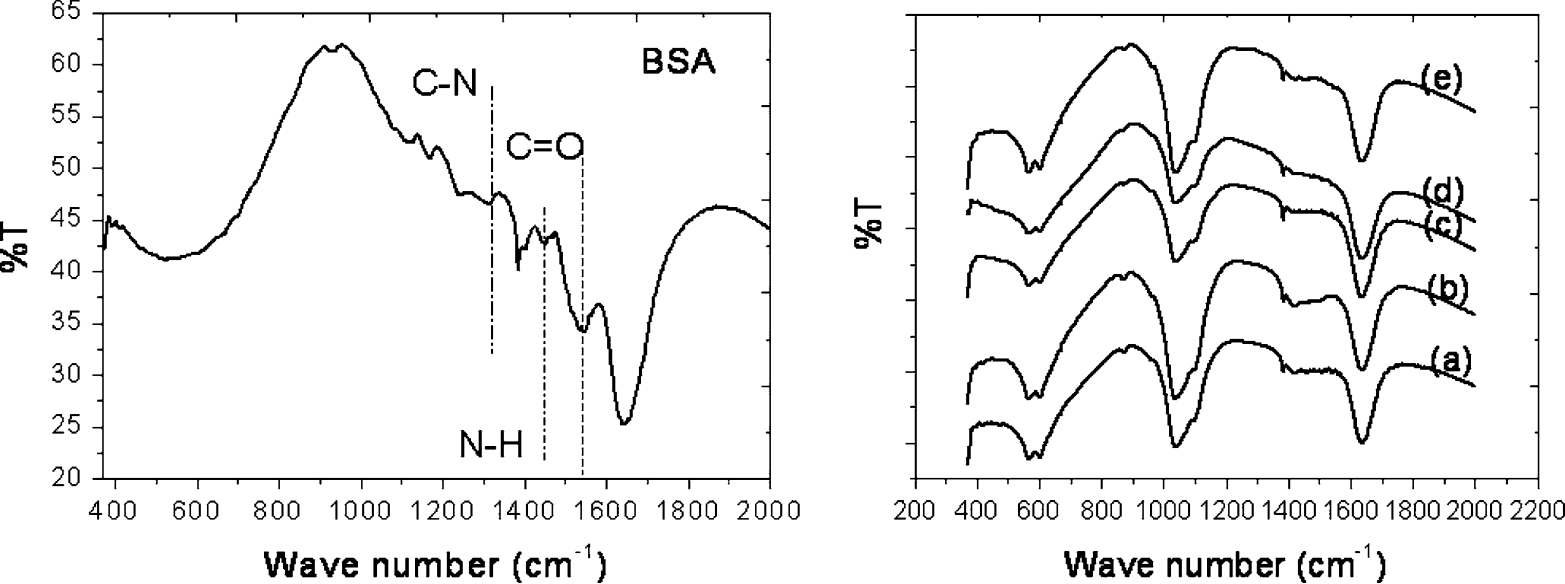
**FTIR spectra of pure BSA protein (*left*), and comparison of BSA protein loading onto the apatite (Ap)-coated citric acid–stabilized cobalt ferrite (CFCA) (CFCA-Ap) surface (*right*) at various concentrations: (*a*) 0.2 mg/ml, (*b*) 0.4 mg/ml, (*c*) 0.6 mg/ml, (*d*) 0.8 mg/ml, and (*e*) 1.0 mg/ml**.

The adsorption of BSA protein by the CFCA-Ap particles is seen in the FT-IR spectrums Figure 6. The IR spectra of pure BSA and of the BSA-loaded CFCA-Ap powders are shown together for comparison. The peaks in the FT-IR spectra of the pure BSA at 1,654 cm^-1^ are due to the C=O stretching mode of amide I; the peak at 1,540 cm^-1^, to the N–H bending mode of amide II and the peak at 1,384 cm^-1^ are due to the C–N stretching mode [39]. The FT-IR spectra of the 0.2–1.0 mg/ml BSA-loaded CFCA-Ap exhibit in addition to the characteristic adsorption bands of apatite, intense bands at 1,088 cm^-1^, at 1,035 cm^-1^ and at 961 cm^-1^. These bands are due to the (PO_4_^3-^) stretching modes. The doublet at 602 and 562 cm^-1^ is due to the (PO_4_^3-^) bending mode. The absorption peaks occurring at around 1,654 cm^-1^ and the weak intensity band at 1,540 cm^-1^ are due to the C=O bond in amide I and the C–H bond in amide II. The fact that there is no obvious changes occurring in the FT-IR spectrum of the CFCA-Ap groups and the presence of the absorption peaks of the BSA in the FT-IR spectrums of the BSA-loaded CFCA-Ap suggests that the BSA adsorption on the CFCA-Ap is a physical adsorption process.

**Figure 7:**
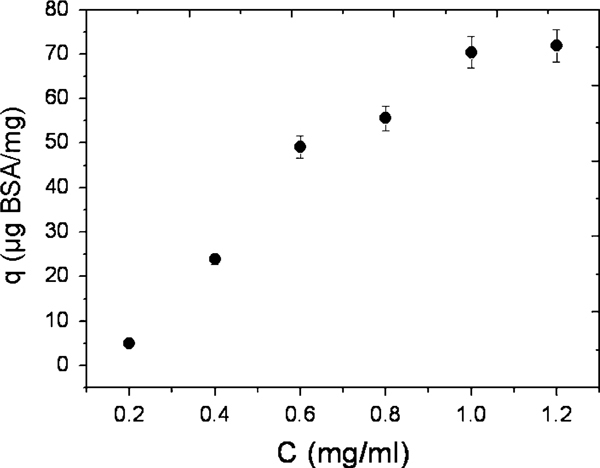
**Adsorption curve of BSA loading onto the apatite (Ap)-coated citric acid–stabilized cobalt ferrite (CFCA) (CFCA-Ap) surface at pH = 7.4 and 37°C**. The data points are expressed as mean ± SD; *n* = 3.

The BSA adsorption was calculated according to the following mass balance before and after equation:

(1) $$q_{\text{e}}=\frac{(C_{0}-C_{\text{e}})V}{W},$$

where *q*_e_ is the BSA adsorption capacity for a unit amount of the particles (mg/g), *C*_0_ is the initial BSA concentration (mg/ml), *C*_e_ is the final or equilibrium BSA concentration (mg/ml), *V* is the volume of BSA solution (ml) (5 ml), and *W* is the weight of the particles added to the solution (mg). The results (Figure 7) show that the amount of bound BSA protein on the particles increases rapidly when the concentration of the BSA is in the range of 0.2–0.6 mg/ml and is lower when the concentration is above 0.6 mg/ml. The amount absorbed reaches a plateau at 1.0 mg/ml.

The in vitro study of BSA protein release from the core–shell CFCA-Ap particles was performed as follows (the concentration of 0.6 mg/ml of BSA protein loaded on CFCA-Ap particles was chosen for study): the BSA-loaded CFCA-Ap particles were placed in 15 ml of phosphate buffer (having pH = 4.0, 6.5 and 7.4) (Fisher Scientific, UK) with the temperature being 37°C. The release of the BSA protein from CFCA-Ap was done by using a shaking bath (SKAKER, Sk-300). The length of time that the BSA protein loaded on CFCA-Aps kept in the PBS buffer was varied (the different time periods used are 0.5, 1, 2, 3, 4, 5, 10, 20, and 30 h). At the end of each period, 3 ml of the supernatant was taken out. The concentration of the BSA protein in each of the 3 ml volumes was determined by using a UV–Vis spectrophotometer (Jenway, model 6405) operating at a wavelength of 280 nm. After each measurement, the supernatant was returned to the system. Assuming that the measurement time is very short compared to the time needed for the BSA protein release, the influence of this return on the release profile is expected to be negligible. The protein content in supernatant is again determined by comparing the measured absorbance to a standard curve for the BSA adsorption, represented by the following equation: *C*_*i*_ = 0.5709A_*i*_ + 0.0654; *R*^2^ = 0.9931, where *C*_*i*_ is the BSA protein concentration and *A*_*i*_ is the UV–Vis absorbance at a wave length of 280 nm.

**Figure 8:**
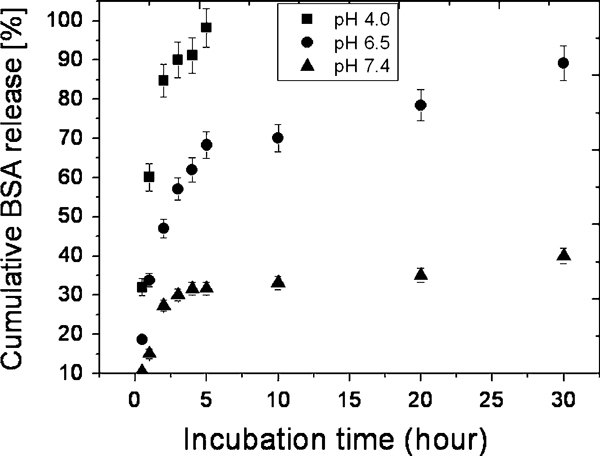
**Release profiles of BSA protein adsorption on apatite (Ap)-coated citric acid–stabilized cobalt ferrite (CFCA) (CFCA-Ap) particles**. The cumulative percent of BSA released versus incubation time for the BSA-loaded CFCA-Ap particles for three pH values (4.0, 6.5, and 7.4) of phosphate buffer saline. The data points are expressed as mean ± SD; *n* = 3.

The concentration of the BSA that is released into PBS (at a pH = 4.0, 6.5 and 7.4) is obtained by comparing the measured absorbance of sample with absorbance on the calibration curve. The BSA release from CFCA-Ap composite particles is determined by plotting the cumulative data against time as illustrated in Figure 8. It reveals that the release rate of BSA from the CFCA-Ap is markedly influenced by the pH value. When the pH is 7.4 or 6.5, the initial release is seen to be lower in the first 5 h than when the pH is 4.0. A burst effect is seen when the pH is at this later value.

The BSA in the burst release when the pH is 4.0 does not only originate from the diffusion of proteins entrapped near the surface but also from within the CFCA particles since the calcium phosphate in the apatite coating on the particles surface dissolves when the environment is acidic. Both phenomena occur simultaneously. This leads to the release of the BSA from the CFCA particles to be very rapid. When the pH is higher, i.e., at pH of 6.5 and 7.4, the release rate is lower since only the proteins trapped at the surface are released. The proteins entrapped within the CFCA-Ap can only be released when they diffuse through the apatite layer and reach the surface. The low permeability of BSA through the porous apatite layer will lead to a low release.

### Analysis of the Adsorption and Release of Proteins by the CFCA-Ap Particles

**Figure 9:**
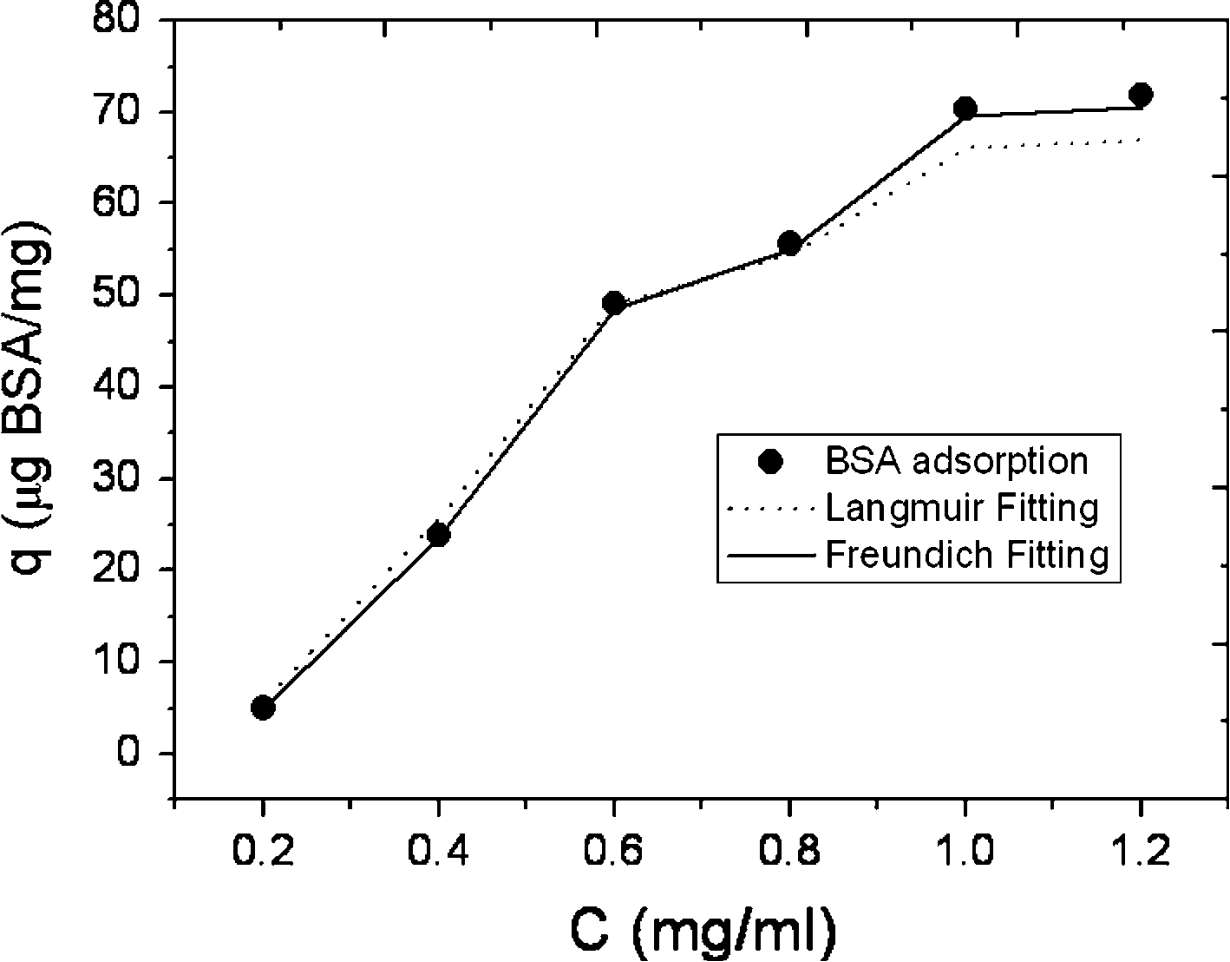
**Comparison of BSA adsorption on apatite (Ap)-coated citric acid–stabilized cobalt ferrite (CFCA) (CFCA-Ap) surface with the Langmuir and Freundlich isotherms at pH 7.4 and 37°C**.

Two equations have been proposed for the relationship between adsorption capacity of the particles and BSA concentration. One of them is the Langmuir adsorption equation [40,41]

(2) $$\frac{1}{q_{\text{e}}}=\frac{1}{q_{\max}}+\frac{1}{q_{\max}bC_{\text{e}}},$$

where *q*_max_ is the maximum adsorption at monolayer coverage (mg/g) and *b* is the Langmuir adsorption equilibrium constant (ml/mg), and reflects the energy of adsorption. The other is the Freundlich adsorption equation [40,41],

(3) $$\ln q_{\text{e}}=\frac{1}{n}\ln C_{\text{e}}+\ln K_{\text{F}},$$

where *K*_F_ and 1/*n* are the Freundlich characteristic constants, whose values indicate whether the absorption leads to a uniform coverage or to a heterogeneous coverage. A heterogeneous coverage would be the case if the value of 1/*n* is close to zero. The Freundlich expression is an empirical equation based on the adsorption on a heterogeneous surface. The relationship between the adsorption capacity and the original BSA concentration has been analyzed using both the Langmuir and the Freundlich isotherms. Figure 9 shows the fitting of BSA adsorption equilibrium on the CFCA-Ap particles at pH 7.4 and 37°C to the Langmuir and the Freundlich isotherms. The calculated Langmuir and Freundlich isothermal adsorption parameters for BSA adsorption are summarized in Table 1. The correlation coefficient (*R*_c_) is 0.993 for the Langmuir and is 0.999 for the Freundlich isotherm. The Freundlich model fits the experimental data better than the Langmuir model when *n* is equal to 1. This implies that the BSA is adsorbed onto the surface of the CFCA-Ap particles in a uniform manner. This suggests that the amount of BSA protein adsorbed onto CFCA-Ap which is regulated by the concentrations of protein in the solution is in the range of 0–1.2 mg/ml.

**Table 1:** Isotherm constants for BSA adsorption onto apatite (Ap)-coated citric acid–stabilized cobalt ferrite (CFCA) (CFCA-Ap) particles

Sample	Langmuir isotherm parameter			Freundlich isotherm parameter		
		
	*b*	***q***_**max**_	***R***_**c**_	***K***_**F**_	*n*	***R***_**c**_
CFCA-Ap	0.685	340	0.993	197.5	1.0	0.999

From the release profile of BSA protein from CFCA-Ap particles, the results (shown in Figure 8) show that the release depends on the pH. Two behaviors are observed: a bust release in the first 5 h especially when the release is occurring in an acidic media (pH 4.0 and 6.5), or a gradual release occurring after 5 h when the pH is at a physiological level of 7.4. It thus appears that the release of BSA from CFCA-Ap particle is controlled by the pH of the surrounding medium. Why this occurs, we need to consider the manner of the release of BSA from apatite [42,43]. First of all, there are positively charged Ca^2+^ ions and negatively charged PO_4_^3-^ ions on the CFCA-Ap particles. The BSA contains negatively charged carboxyl (–COO^-^) and positively charged amino (–NH_3_^+^) as side groups. The interaction between the opposite charges on the apatite with the BSA inhibits the release of the protein until the calcium phosphate of apatite is dissolved.

**Figure 10:**
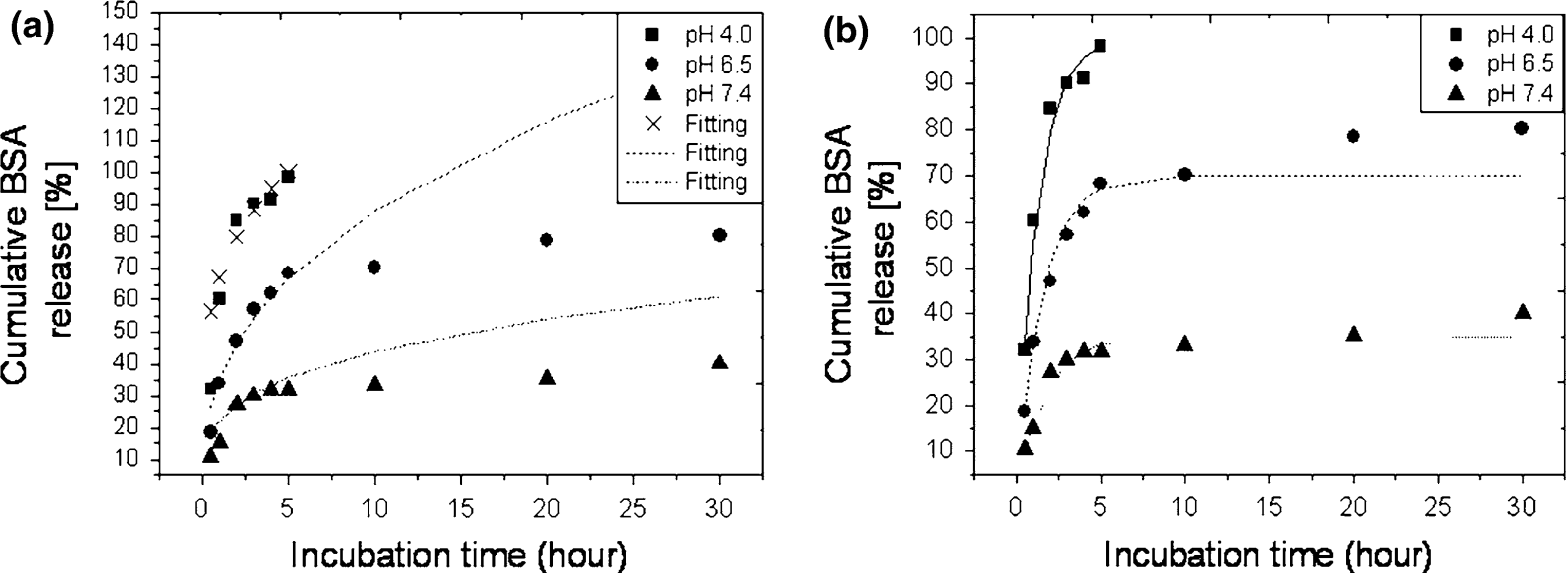
**Fit of the release data to kt^*n*^ (a) and Fit of the release data to *φ*_o_(1 - exp(-*k*_b_*t*)) (b)**. The observed values and the fitted values to kt^*n*^ when the pH values of the buffer solution are 4.0, 6.5, and 7.4. The values of the *k* and *n* are (67.215 h^-1^,0.25) for pH 4.0, (35.031 h^-1^, 0.40) for pH 6.5 and (22.184 h^-1^, 0.30) for pH 7.4, respectively. For the observed values and the fitted values to *φ*_o_(1 - exp(-*k*_b_*t*)) of the *φ*_o_ and *k*_b_, which lead to the best fits are (100.00, 0.805 h^-1^), (70.430, 0.659 h^-1^) and (35.417, 0.605 h^-1^), respectively, for the three pH values of 4.0, 6.5, and 7.4.

The dissolution of biomolecules held together by electrostatic force is commonly described by the Peppas's model [44]:

(4) $$\frac{M_{t}}{M_{0}}=kt^{n},$$

where *M*_*t*_ and *M*_0_ are the concentrations of the protein (part of the biomolecule which in the present case is BSA) released into a medium up to time *t* and the initial concentration of protein on the particles. *M*_*t*_/*M*_0_ is the fractional concentration of the released protein. The Peppas's model was developed for water-soluble release from a polymeric device. It has been applied to porous ceramic materials even though the mechanism of release is unknown. It is in good agreement with the release kinetics of biomolecules from devices that have a fast dissolution rate compared to the diffusion times. We have applied a regression analysis to fit Peppas's model, Eq. 4, to the data given on Figure 8. The results of these fits for the different pH environments are shown in Figure 10a. The correlation coefficients, *R*_c_, are 0.957, 0.883, and 0.868 for the pH 4.0, 6.5, and 7.4 data, respectively. While the fits to the data for the first five-hour period are quite good, the fits after the first 5 h are poor, especially the release of BSA in a mild acidic medium (pH 6.5) and in the physiological pH level (pH 7.4).

Ignoring the poor fits of data to Eq. 4 for times after 5 h, we have used Eq. 4 to determine the values of *n* for the different pH level media. The release exponential *n* depends on the transport mechanism and on the geometry of the carrier. The regression analyses give the values of *n* of 0.25, 0.40, and 0.30 when the pH is 4.0, 6.5, and 7.4, respectively. The value of *n* between 0.25 and 0.40 indicates that the transport process for the BSA release is Fickian diffusion [44]. To understand the overall release behavior in the three pH media, we have

$$k_{\text{pH}=4.0}(67.215{\text{ h}}^{-1})>k_{\text{pH}=6.5}(35.037{\text{ h}}^{-1})>k_{\text{pH}=7.4}(22.184{\text{ h}}^{-1})$$

This indicates that the release rate of BSA is pH sensitive and that the release constant *k* decreases with increasing pH. The higher rate of release in acidic environment results from abrupt morphological changes caused by dissolution of the calcium phosphate of apatite. This implies that the BSA release is mainly controlled by the particles dissolution which in turn leads to the release of tightly bound BSA.

To obtain a better fit to the data observed over a longer period, Batycky et al. [45] suggested that the relationship

(5) $$\ln\left(1-\frac{M_{t}}{M_{0}}\right)=-k_{\text{b}}t$$

be used instead. This relationship suggests that the release of BSA from CFCA-Ap is a first-order release process. A regression analysis of the observed data using Eq. 5 leads to the fits shown in Figure 10b. Looking at these fits, we see that the fits are much better than the fits to Peppas's model (see Figure 10a). The correlation coefficients are now 0.989, 0.974, and 0.973, for pH of 4.0, 6.5, and 7.4, respectively. These results indicate that the first-order release model is a better model for describing the release of BSA from CFCA-Ap matrix than is the Peppas's model. The correlation coefficients, *R*_c_, and the rate constants, *k*_b_, generated by the regression analyses of both model are listed in Table 2.

**Table 2:** Release characteristic parameters of release profiles of BSA adsorption on apatite (Ap)-coated citric acid–stabilized cobalt ferrite (CFCA) (CFCA-Ap) particles

pH	***Y* = *kt***^***n***^**; *y* = *M***_***t***_**/*M***_**0**_			***Y* = *φ***_**b**_**(1 - exp(-*k***_**b**_***t*)); *y* = *M***_***t***_**/*M***_**0**_		
		
	***K* (h**^**-1**^**)**	*n*	***R***_**c**_	***φ***_**b**_	***k***_**b **_**(h**^**-1**^**)**	***R***_**c**_
4.0	67.215	0.25	0.957	100.00	0.805	0.989
6.5	35.031	0.40	0.883	70.430	0.659	0.974
7.4	22.184	0.30	0.868	35.417	0.605	0.973

*φ*_b_ is the fraction of drug released through initial burst

## Conclusion

BSA-loaded CFCA-Ap particles were synthesized through the biomineralization of Co-ferrite particles in a 1.5SBF solution after which BSA loaded on. XRD and FT-IR spectra indicated that the coating on the CFCA-Ap particles consisted of apatite mineral. The core–shell structure of the CFCA-Ap particles was observed by TEM. The sizes of the particles were in the range of 400–1,200 nm. The CFCA-HAp particles have a good capacity for adsorption of BSA. The adsorption is better described by a Freundlich isotherm than by a Langmuir isotherm. It is found that the release of BSA by the CFCA-Ap particles is pH dependent. The release of the BSA from the CFCA-Ap particles is greatly accelerated when the pH of the medium is lowered. These results suggest that CFCA-Ap particles can be useful for the sustained release of protein or other biomolecules (e.g., DNA), in an acidic media. This would be especially important if the acidity is due to a bacterial infection or inflammation.
